# HIV‐1 exposure and immune activation enhance sexual transmission of Hepatitis C virus by primary Langerhans cells

**DOI:** 10.1002/jia2.25268

**Published:** 2019-04-01

**Authors:** Bernadien M Nijmeijer, Ramin Sarrami‐Forooshani, Gaby S Steba, Renée RCE Schreurs, Sylvie M Koekkoek, Richard Molenkamp, Janke Schinkel, Peter Reiss, Matthijs L Siegenbeek van Heukelom, Marc van der Valk, Carla MS Ribeiro, Teunis BH Geijtenbeek

**Affiliations:** ^1^ Department of Experimental Immunology Amsterdam Infection and Immunity Institute Amsterdam University Medical Centers University of Amsterdam Amsterdam The Netherlands; ^2^ Department of Medical Microbiology Clinical Virology Laboratory Amsterdam University Medical Centers University of Amsterdam Amsterdam The Netherlands; ^3^ Department of Global Health Amsterdam University Medical Centers, and Amsterdam Institute for Global Health and Development Amsterdam University Medical Centers HIV Monitoring Foundation Amsterdam The Netherlands; ^4^ Division of Infectious Diseases Department of Internal Medicine Amsterdam Infection and Immunity Institute Amsterdam University Medical Centers University of Amsterdam Amsterdam The Netherlands; ^5^ Department of Dermatology Amsterdam University Medical Centers University of Amsterdam Amsterdam The Netherlands

**Keywords:** HIV‐1, Langerhans Cells, Hepatitis C virus, Immune activation, Viral transmission, Men‐who‐have‐Sex‐with‐Men

## Abstract

**Introduction:**

The significant rise in incidence of Hepatitis C virus (HCV) infection among men‐who‐have‐sex‐with‐men (MSM) living with HIV‐1 suggests that HCV under specific circumstances is transmitted via sexual contact. During sexual transmission HCV has to cross the epithelial barrier to either directly enter the blood stream or indirectly via mucosal immune cells. However, the mechanisms of sexual transmission of HCV remain unclear. We investigated the role of Langerhans cells (LCs) in HCV susceptibility during sexual contact as LCs are among the first cells in mucosal tissues to encounter invading viruses.

**Methods:**

We investigated the phenotype of primary LCs in anal biopsies from MSM living with HIV‐1. To investigate the role of primary LCs in HCV infection and transmission, we have used both isolated primary skin LCs and the *ex vivo* tissue transmission model.

**Results:**

Our data identified an important role for mucosal LCs in facilitating HCV transmission after HIV‐1 exposure or immune activation. LCs were detected within mucosal anal tissues obtained from HIV‐1 positive MSM biopsies. In order to perform functional studies, we used primary LCs from skin, which have a similar phenotype as mucosal LCs. Immature LCs were neither infected nor transmitted HCV to hepatocytes. Notably, exposure to HIV‐1 significantly increased HCV transmission by LCs in the *ex vivo* transmission model. HIV‐1 replication was crucial for the increased HCV transmission as HIV‐1 inhibitors significantly reduced HIV‐1‐induced HCV transmission. Moreover, tissue immune activation of LCs also increased HCV transmission to target cells.

**Conclusions:**

Thus, our data strongly indicate that HIV‐1 or immune activation in MSM leads to capture of HCV by mucosal LCs, which might facilitate transmission to other cells or allow entry of HCV into the blood. This novel transmission mechanism by LCs also implicates that the activation state of LCs is an important cellular determinant for HCV susceptibility after sexual contact.

## 1 Introduction

Globally, 115 million people are infected with Hepatitis C virus (HCV) whereas 37 million people are infected with HIV‐1, and approximately two million infected with both HIV‐1 and HCV 1. Hepatitis C virus infection has been recognized as an emerging infection in man‐who‐have‐sex‐with‐man (MSM) 2. Studies indicated that HIV‐1 status is an important factor for sexually acquired HCV 3, 4, 5, 6 but recent studies suggest that sexual transmission also occurs among HIV‐uninfected MSM 7, 8. Thus, HCV is sexually transmitted but the mechanisms remain unclear. Individuals living with HIV‐1 are less likely to spontaneously clear HCV infection during acute infection, have higher HCV viral loads, and experience more rapid liver fibrosis progression than those without HIV‐1 infection 9, 10, 11. Moreover, mucosal tissue damage as well as sexual practices leading to rectal bleeding are factors that can enhance HCV susceptibility after sexual contact 12. Hepatitis C virus infection in individuals living with HIV‐1 has a major impact as in the developed world, end‐stage liver disease is a leading cause of death among HIV‐1 positive individuals 13. Although treatment with directly acting antivirals (DAA) against HCV is very effective in clearing HCV 14, MSM living with HIV‐1 remain at high risk of reinfection 4, 15, 16. These recent studies suggest that HCV is sexually transmitted within MSM 17, whereas sexual transmission of HCV within heterosexual HIV‐1 infected or uninfected individuals is rare 18. Apart from intravenous drug use, factors for acquiring HCV are receptive unprotected anal intercourse and other sexual risk behaviour 19, 20. It has also been shown that HCV is shed into the rectum in HIV‐1 positive men with HCV infection 21. These studies strongly suggest that the rectal mucosa is the primary entry site for HCV entry in MSM after sexual contact, and that unknown mechanisms allow changes within the mucosa that increase susceptibility to HCV. HIV‐1 infection might directly affect the activation of mucosal immune cells, or cause defective mucosal barrier 22, whereas in uninfected MSM other sexually transmitted infections (STIs) might change the mucosal barrier allowing HCV entry.

Interestingly, how HCV after entry into mucosa reaches the blood and liver remains unclear. Dendritic cell (DC) subsets play a role in sexual transmission of viruses such as HIV‐1 across mucosal tissues 23. Several studies have shown that submucosal DCs are able to capture HCV and transmit the virus to neighbouring human liver cells 24, 25. Mucosal Langerhans cells (LCs) have been identified in human sigmoid colon and rectal mucosal tissues 26, therefore LCs could be among the first cells that encounter HCV upon sexual transmission in MSM. Little is known about the function of mucosal LCs in HCV susceptibility. Here we show an important role for mucosal LCs in HCV transmission. We identified mucosal LCs in anal biopsies from MSM living with HIV‐1. As the cell numbers are very low we used primary LCs from skin to investigate their function in HCV transmission. Our data strongly suggest that under normal conditions LCs are refractory to HCV but upon activation or HIV‐1 infection, LCs capture HCV. This might allow either transmission to other cells such as lymphocytes that subsequently migrate into the blood or allow the virus to survive long enough to enter the blood. Thus efficient capture and retention of HCV in mucosal tissues by LCs might increase HCV susceptibility in MSM.

## 2 Methods

### 2.1 Antibodies and reagents

The following reagents were used: tripalmitoylated‐lipopeptide Pam3CSK4 (5 μg/mL) (Invivogen, San Diego, CA, USA), recombinant human TNF (1 μg/mL) (R&D Systems, Minneapolis, MN, USA). The following antibodies were used (all anti‐human): CD3‐AF700, CD3‐PerCP (BD Biosciences, San Jose, CA, USA), CD19‐FITC, CD20‐FITC, CD56‐FITC, (Thermo Fisher Scientific Waltham, MA, USA), CD45‐V500, (BD Horizon, USA), CD1a‐APC (BD Biosciences, San Jose, CA, USA), CD207‐PE (Beckman Coulter, Indianapolis, IN, USA), DC‐SIGN‐FITC (R&D Systems) and anti‐HIV‐1 p24, KC57‐RD1‐PE, (Beckman Coulter). The following reagents were obtained through the NIH AIDS Reagent Program, NIAID: Zidovudine (10 μmol/L), Raltegravir (100 nmol/L) and Indinavir (1 μmol/L).

The following plasmids were provided by Dr. Takaji Wakita at Tokyo Metropolitan Institute of Neuroscience: Genotype 2a HCV genomic RNA clone pJFH1 (APP1025) 27. pJFH1‐AM120‐Rluc was provided by Dr. Curt Hagedorn at University of Utah, Salt Lake City, USA. pNL4.3.Luc_RΔenv provided by Dr. N.R. Landau 28, pHCV_H77_E1_E2(AF009606) Dr. Joe Grove (Addgene) 29.

### 2.2 Cell lines

Huh7.0 (human hepatocellular carcinoma) cell line was obtained from the Health Science Research Resources Bank (JCRB0403; Osaka, Japan). Cells were maintained in Dulbecco modified Eagle medium (Gibco Life Technologies, Gaithersburg, MD) containing 10% fetal calf serum (FCS) and penicillin/streptomycin. Huh7.5 cells were provided by Dr. Charles M. Rice 30. Medium was supplemented with 1 mmol/L Hepes buffer (Gibco Life Technologies). MUTZ‐LCs were differentiated from CD34^+^ human AML cell line MUTZ3 progenitors in the presence of GM‐CSF (100 ng/mL; Thermo Fisher Scientific), TGF‐β (10 ng/mL; R&D Systems) and TNF‐α (2.5 ng/mL), R&D Systems) and cultured as described before 31.

### 2.3 HIV‐1 and HCV virus stocks

The following virus stocks were obtained through the NIH AIDS Reagent Program, division of AIDS, NIAID: HIV‐1 JR‐CSF Virus from Dr. Irvin Chen 32, 33, HIV‐1 SF162 Virus from Dr. Jay Levy 34. HIV‐1 virus stocks were propagated on PHA‐stimulated human PBMCs. Produced HIV‐1 viruses were quantified by p24 ELISA (Perkin Elmer Life Sciences, Boston, MA, USA) and titrated using the indicator cells TZM‐bl (John C. Kappes, Xiaoyun Wu, Birmingham, Alabama, USA and Tranzyme Inc., the NIH AIDS Reagent Program, division of AIDS, NIAID) 35. For single‐round infection assay, human embryonic kidney 293T/17 cells (ATCC, CRL‐11268) were co‐transfected with pNL4.3.Luc_RΔenv, containing firefly luciferase gene at the *nef* position (1.35 μg) and pHCV_H77_E1_E2(AF009606) (0.6 μg). Transfection was performed in 293T/17 cells using genejuice (Novagen, Merck millipore, Burlington, MA, USA) transfection kit according to the manufacturer's protocol. At day 2, pseudotyped HCV was harvested and filtered over 0.45 μm nitrocellulose membrane (SartoriusStedim, Gottingen, Germany). JFH1‐AM120‐Rluc In vitro transcribed RNA was generated according to the manufacturer's instructions (Ambion MEGAscript‐kit; Thermo Fisher Scientific, Waltham, MA, USA) and electroporated into Huh7.5 cells as previously described 36. Virus particles were harvested on day 8 and, TCID50s were determined. The TCID50 of HCV ranged from 2 × 10^3^ to 4 × 10^3^.

### 2.4 HIV‐1 anal biopsies analysis

Anal biopsies from HIV‐1 positive MSM under cART 37 were taken from the anal transformation zone following the “2016 IANS International guidelines for practice standard” 38. Samples were minced and incubated for one hour at 37°C with IMDM (Thermo Fischer Scientific) containing 1 mg/mL Collagenase D (0.15 U/mg; Roche, Switzerland) 1% FCS and 1000 U/mL DNAse type I (Roche Applied Sciences, Mannheim, Germany), pushed through a 70 μm single‐cell strainer (Falcon, VWR, United Kingdom). Single‐cell suspension was washed, stained with monoclonal antibodies (all anti‐human), 30 minutes at 4°C. Cells were washed with PBS, resuspended in 1× stabilizing fixative (BD Biosciences) 39. Flowcytometric analyses were performed on LSR Fortessa (BD) 24 hours after fixation. Data were analysed using FlowJo vX.0.7 software (TreeStar Inc, Ashland, OR, USA).

### 2.5 Ex vivo model and primary LC isolation

Epidermal sheets were prepared as described previously 40, 41. Briefly, skin‐grafts were obtained using a dermatome (Zimmer Biomet, Indianapolis, IN, USA). After incubation with Dispase II (1 U/mL; Sigma Aldrich, Saint Louis, Missouri, USA), epidermal sheets were separated from dermis, washed, cut in 1 cm^2^ and cultured in Iscoves Modified Dulbeccos's Medium (IMDM; Thermo Fischer Scientific) supplemented with 10% FCS, gentamycine (20 μg/mL; Centrafarm, Etten‐Leur, The Netherlands), pencilline/streptomycin (10 U/mL and 10 μg/mL respectively; Invitrogen). LC‐enriched epidermal single‐cell suspensions were generated as described before 40, 41. Briefly, epidermal sheets were incubating in PBS containing DNase I (20 units/mL; Roche Applied Science, Mannheim, Germany) and trypsin 0.05% (Beckton Dickinson, USA). Single‐cell suspension was layered on Ficoll gradient (Axis‐shield) and immature LCs were purified using CD1a microbeads (Miltenyi Biotec, Bergisch Gladbach, Germany). LCs were routinely 85% to 98% pure and expressed high levels of Langerin and CD1a 23. Mature LCs were generated as described before 40. Briefly, epidermal sheets were cultured in IMDM (Thermo Fischer Scientific) supplemented with 10% FCS, gentamycine (20 μg/mL; Centrafarm, Etten‐Leur, The Netherlands), pencilline/streptomycin (10 U/mL and 10 μg/mL respectively; Thermo Fisher Scientific) for three days and mature LCs were harvested.

### 2.6 Ex vivo transmission and co‐culture

Epidermal sheets were incubated with Raltegravir (100 Nmol/L) or Indinavir (1 μmol/L), and after two hours exposed to JRCSF (100 μL/sheet, TCID50 of 38 x 10^3^, determined in TZM‐Bl cells) for 24 hours and subsequently to replicative HCV or pseudotyped HCV for another 24 hours. After 48 hours cells were harvested, extensively washed to remove unbound virus and co‐cultured with Huh7.0 or Huh7.5 for five days at 37C. Luciferase activity (relative light units (R.L.U.)) was measured using the Luciferase assay system (Promega, Madison, WI, USA) according to manufacturer's instructions.

### 2.7 Strand‐specific quantitative real‐time PCR

Specific amplification of HCV minus strand RNA was performed as previously described 42, modified for Taqman realtime PCR. cDNA of the HCV minus strand was generated by reverse transcription using thermoscript (Thermo Fisher Scientific) and primer tag‐RC1 (5′‐*ggccgtcatggtggcgaataa*GTCTAGCCATGGCGTTAGTA‐3′) according to manufacturer instructions. Subsequently the tagged minus strand cDNA was amplified and detected by realtime Taqman PCR using a tag‐specific primer, RC21 (5′‐CTCCCGGGGCACTCGCAAGC‐3′), and FAM‐labelled Taqman probe HCV‐5UTR (5′‐CTCCCGGGAGAGCCATAGTGGTCTGCG‐3′) using LC480 Probesmaster (Roche Applied Science) according to manufacturer instructions. PCR conditions were as follows: two minutes at 50°C and ten minutes at 95°C, followed by 45 cycles each consisting of fifteen seconds at 95°C and one minutes at 60°C. Ct values above 36 were considered background, based on negative control experiments.

### 2.8 HCV binding and uptake assay

Mature LCs were exposed to pseudotyped HCV for 5h at 4C or 37C, harvested, trypsinized, washed, lysed and measure for p24 ELISA (Perkin Elmer Life Sciences) according to manufacturer instructions.

### 2.9 Cell sorting

Mature LCs were sorted using CD1a‐APC and CD3‐PerCP and analysed on a FACS Aria (BD). CD3 negative and CD1a positive mature LCs were exposed to pseudotyped HCV, after 24 hours cells were harvested, extensively washed and co‐cultured with Huh7.5 for five days at 37°C.

### 2.10 Statistics

Two‐tailed Student's *t*‐test for paired observations (differences between different donors) or unpaired observation (differences within the same donor). Statistical analyses were performed using GraphPad Prism 7 software and significance was set at *p<0.05, **p < 0.01***p < 0.001****p < 0.0001.

### 2.11 Study approval

HIV‐1 anal biopsy samples for this study were collected after written informed consent was given by the participant. Sample collection was approved by the local ethical committee under project/reference number: 05/031. Human skin tissue was obtained from healthy donors undergoing corrective abdominal surgery in accordance with our institutional guidelines. This study was approved by the Medical Ethics Review Committee of the Academic Medical Center (W15_089 # 15.0103). Biopsy samples were collected from November 2016 until May 2017. The whole of this study was conducted from January 2015 until January 2018.

## 3 Results

### 3.1 Langerhans cells are present in anal mucosal biopsies from HIV‐1 infected MSM

**Figure 1:**
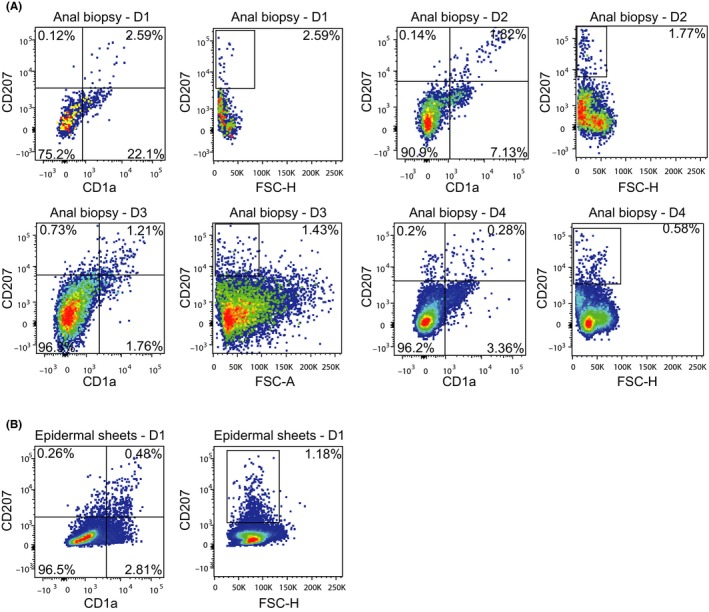
Langerhans cells are present in anal mucosal biopsies from HIV‐1 infected MSM **(A)** Single cell suspensions of anal biopsies from HIV‐1 infected individuals were stained with antibodies against CD45, CD19, CD20, CD56, CD3, CD1a and CD207 and analysed by flow cytometry. The percentage of cells present is depicted in the upper‐right corner of the dot plots. Four representative donors out of six donors is depicted. **(B)** Single cell suspensions of epidermal sheets were stained with antibodies against CD1a and CD207 and analysed by flow cytometry. One representative donor is depicted. D1, Donor 1; D2, Donor 2; D3, Donor 3; D4, Donor 4.

Here, we investigated the presence of LCs in anal mucosa. Single‐cell suspensions of biopsies from MSM living with HIV‐1 were analysed by flow cytometry (Figure S1A,B) 39. Notably, a LC population based on the LC‐markers CD1a and Langerin (CD207) was detected in multiple donors. The absolute number of LCs ranged from 80 to 120 in a 5 by 5 mm biopsy (Figure 1A). Interestingly, the phenotype as well as total number of LCs from colonic mucosa was similar to those observed in epidermal sheets (Figure 1B). Moreover, mucosal LCs did not express DC‐SIGN, similar as skin‐derived LCs (Figure S2A,B,C), further indicating that these mucosal cells are LCs. These data suggest that anal mucosa contains similar amount of LCs as observed in epidermis, which strongly indicates that anal mucosal LCs might have an important function in defence against pathogens and might be an important target for HCV upon infection.

### 3.2 Immature, mature and activated Langerhans cells do not become infected by HCV

**Figure 2:**
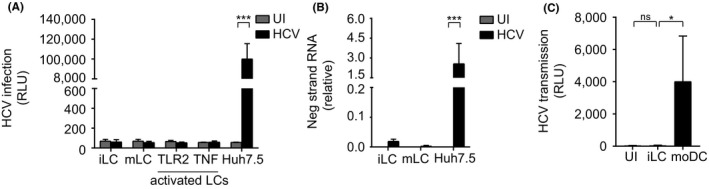
Immature, mature and activated Langerhans cells do not become infected by HCV **(A)** Immature and mature LCs were isolated and stimulated with Pam3CSK4 (TLR2) (5 μg/mL) and TNF (1 μg/mL), after two hours, cells were infected with infectious HCV (JFH1) for six days and analysed for luciferase reporter activity. Error bars are the mean ± SD of n=2 (immature LCs) and n=4 (mature LCs) donors measured in duplo and n=3 (Huh7.5 cell line) in quadruplo. ***p < 0.001, by two‐tailed, unpaired Student's *t*‐test. **(B)** Immature and mature LCs were isolated and infected with infectious HCV (JFH1) for six days and analysed for negative strand RNA by real‐time PCR normalized to beta‐globuline. Error bars are the mean ± SD of n=3 (immature LCs), n=4 (mature LCs) donors measure in duplo, and n=3 (Huh7.5 cell line) in triplo and quadruplo. ***p < 0.001, by two‐tailed, unpaired Student's *t*‐test. **(C)** Immature LCs and moDCs were isolated and infected with infectious HCV (JFH1) for 24 hours, extensively washed, co‐cultured with huh7.5 cells and analysed for luciferase reporter activity. Error bars are the mean ± SD of n=3 (immature LCs) donors measured in duplo and n=3 (moDCs) measured in triplo. *p < 0.05, ns, not significant, by two‐tailed, unpaired Student's *t*‐test. HCV, Hepatitis C virus; iLC, immature langerhans cell; mLC, mature langerhans cell; moDC, monocyte derived dendritic cell; UI, uninfected.

We investigated whether HCV infects primary immature LCs using a replicative HCV genotype 2a strain containing a luciferase reporter gene (JFH1‐AM120‐Rluc), that expresses luciferase upon replication 36. Due to the low amounts of LCs from biopsies, LCs were isolated from human skin epidermis 40. Both immature and mature LCs were exposed to replicative HCV for six days or left uninfected. Notably, neither immature nor mature LCs were infected by HCV (Figure 2A,B). Moreover, activation of mature LCs by TLR ligands did not induce infection of LCs by HCV (Figure 2A). Furthermore, no replication intermediates were detected in both immature and mature LCs by negative strand PCR, whereas the hepatocyte cell‐line Huh7.5 was efficiently infected (Figure 2B). These data strongly indicate that neither immature, mature nor activated LCs are infected by HCV. Several studies showed that immature submucosal DCs are able to transmit HCV via DC‐SIGN 24, 25. Therefore we investigated whether immature LCs could also transmit HCV. Both Immature LCs and DCs were incubated with infectious HCV (JFH1‐AM120‐Rluc) for 24 hours, and after extensive washing co‐cultured with Huh7.5 cells. In contrast to DCs, co‐culture of HCV‐treated immature LCs with hepatocyte cell‐line did not result in HCV infection of the hepatocytes (Figure 2C), strongly suggesting that immature LCs in contract to immature DCs are not able to retain HCV for transmission to target cells.

### 3.3 HIV‐1 replication enhances HCV transmission by LCs ex vivo

**Figure 3:**
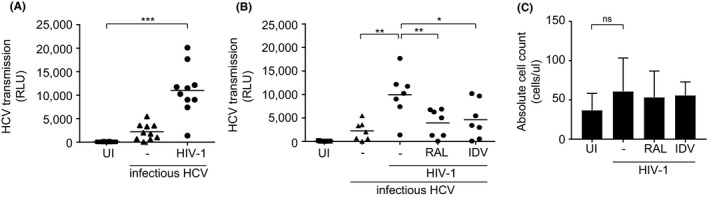
HIV‐1 replication enhances HCV transmission by LCs ex vivo **(A‐B)** Epidermal sheets were floated on medium in a 24‐well plate and exposed to either HIV‐1 (JRCSF) or HIV‐1 (SF162) for 24 hours and subsequently exposed to infectious HCV (JFH1‐AM120‐Rluc) for another 24 hours. Emigrated cells were harvested, extensively washed, co‐cultured with huh7.5 cells and analysed for luciferase reporter activity. **(A)** Each dot represents 1 donor. Horizontal bars are the means of n=10 donors measured in triplo or quadruplo. ***p < 0.001 by two‐tailed, paired Student's *t*‐test. **(B)** Epidermal sheets were pre‐exposed to HIV‐1 replication inhibitors Raltegravir or Indinavir for two hours. Each dot represents 1 donor. Horizontal bars are the means of n=7 donors measured in quadruplo. *p < 0.05, **p < 0.01, by two‐tailed, paired Student's *t*‐test. **(C)** Epidermal sheets were pre‐exposed to HIV‐1 replication inhibitors Raltegravir or Indinavir for two hours, exposed to either medium only or HIV‐1 (JRCSF) for 48 hours. Emigrated LCs were harvested, stained with an antibody against CD1a and absolute cell count beads and analysed by flow cytometry. Error bars are the mean ± SD of n=2 donors measured in duplo. ns, not significant, by two‐tailed, unpaired Student's *t*‐test. HCV, Hepatitis C virus; IDV, Indinavir; RAL, Raltegravir; UI, uninfected.

As HCV transmission occurs in MSM living with HIV‐1, we investigated the effect of HIV‐1 on HCV transmission in the *ex vivo* tissue transmission model 23. *Ex vivo* tissues were exposed to HIV‐1 for 24 hours and subsequently exposed to HCV (JFH1–AM120‐Rluc) for another 24 hours. LCs migrated from these tissues had a mature phenotype 23. After 48 hours emigrated LCs were harvested, extensively washed and co‐cultured with Huh7.5 cells, and infection was measured by luciferase activity. Hepatitis C virus exposure to tissue *ex vivo* resulted in low levels of HCV transmission by LCs to hepatocytes. Strikingly, HIV‐1 pre‐exposure to tissues *ex vivo* significantly increased HCV transmission of LCs to hepatocytes (Figure 3A). These data strongly suggest that HIV‐1 exposure allows LCs to capture infectious HCV and retain the virus for transmission to target cells. To further investigate whether HIV‐1 infection is necessary for LCs to retain and transmit infectious virus, we treated *ex vivo* tissue with HIV‐1 integrase inhibitor Raltegravir and protease inhibitor Indinavir prior to HIV‐1 exposure. Notably, treatment of the tissue with the different anti‐retroviral inhibitors abrogated HCV transmission by HIV‐1‐exposed LCs to target cells (Figure 3B). HIV‐1 infection of LCs was blocked by the different HIV‐1 inhibitors (Figure S3). These data strongly suggest that active HIV‐1 replication is required for the observed induction of HCV transmission. To investigate whether HIV‐1 affects the number of emigrated cells we quantified the migration of LCs from *ex vivo* epidermal sheets by flow cytometry. *Ex vivo* tissues were exposed to either medium, HIV‐1 or Indinavir or Raltegravir and HIV‐1 for 48 hours, harvested and counted by flow cytometry. There was no significant difference in absolute cell counts between unexposed and HIV‐1 exposed samples in presence or absence of inhibitors (Figure 3C). Therefore, these data suggest that HIV‐1 replication in the *ex vivo* tissue model affects the function of LCs facilitating HCV capture and transmission by LCs.

### 3.4 Mature and HIV‐1‐infected LCs retain infectious HCV for transmission

**Figure 4:**
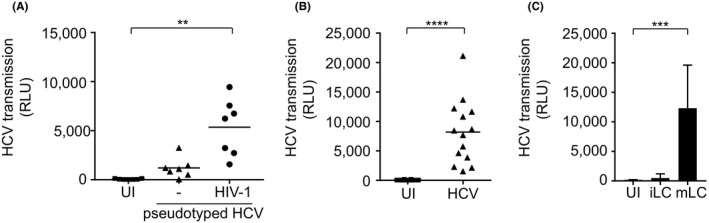
Mature and HIV‐1‐infected LCs retain infectious HCV for transmission **(A)** Epidermal sheets were exposed to HIV‐1 (JRCSF) for 24 hours and subsequently exposed to pseudotyped HCV (HIV‐1 NL4.3Δenv pseudotyped with HCV env glycoproteins E1 and E2) for another 24 hours, extensively washed, co‐cultured with huh7.5 cells and analysed for luciferase reporter activity. Horizontal bars are the means of n=7 donors measured triplo and quadruplo. **p < 0.01, by two‐tailed, paired Student's *t*‐test. **(B)** Mature LCs were exposed to pseudotyped HCV (HIV‐1 NL4.3Δenv pseudotyped with HCV env glycoproteins E1 and E2) for 24 hours, extensively washed, co‐cultured with huh7.5 cells and analysed for luciferase reporter activity. Horizontal bars are the means of n=14 donors measured in duplo, triplo and quadruplo. ****p < 0.0001, by two‐tailed, paired Student's *t*‐test. **(C)** Immature and mature LCs from the same donor were isolated, exposed to pseudotyped HCV (HIV‐1 NL4.3Δenv pseudotyped with HCV env glycoproteins E1 and E2) for 24 hours, extensively washed, co‐cultured with huh7.5 cells and analysed for luciferase reporter activity. Error bars are the mean ± SD of n=3 donors measured in triplicates. ***p < 0.001, by two‐tailed, paired Student's *t*‐test. HCV, Hepatitis C virus; iLC, immature Langerhans cell; mLC, mature Langerhans cell; UI, uninfected.

As we did not observe any infection of mature LCs by HCV, we investigated whether HIV‐1 infected LCs transmit HCV independent of replication using HCV pseudotyped single‐round virus, LCs exposure to HCV pseudotyped viruses *ex vivo* resulted in low levels of HCV transmission but, HIV‐1 exposure significantly increased transmission of HCV pseudotyped viruses by LCs (Figure 4A). These data strongly indicate that HIV‐1 infection increases the ability of LCs to retain infectious HCV viruses, resulting in HCV transmission independent of infection.

Next, we investigated whether the maturation of LCs affects HCV transmission. Mature LCs were isolated from a three‐day culture of epidermal sheets 40. These emigrated LCs had an mature phenotype as shown by the higher expression levels of co‐stimulatory molecules CD80 and CD86, and decreased Langerin expression, compared to immature LCs 41. Mature LCs were exposed to HCV pseudotyped virions for 24 hours, washed and co‐cultured with Huh7.5 cells for 5 days. Strikingly, mature in contrast to immature LCs transmitted HCV to target cells (Figure 4B). Moreover, sorted CD3‐negative, CD1a‐positive LCs as well as CD34‐derived MUTZ‐LCs transmitted HCV to target cells (Figure S4A,B). These data exclude that LCs and not contaminating T cells transmit HCV. To further compare the impact of maturation on LCs, we isolated immature and mature LCs from the same donor and performed a transmission assay. Mature but not immature LCs from the same donors efficiently transmitted HCV to Huh7.5 cells (Figure 4C). Furthermore, mature LCs efficiently bound HCV, which was rapidly internalized as trypsin treatment did not diminish HCV uptake (Figure S5). Thus, the maturation state alters the transmission capacity of LCs leading to virus retention for transmission. These findings indicate that not only HIV‐1 exposure, but also LC maturation by immune activation induces HCV transmission by LCs.

## 4 Discussion

The incidence of HCV is on the rise and re‐infection rates are high in MSM living with HIV‐1 15, 16. Several studies suggest that HCV is transmitted via sexual contact within this population 3, 4, 5, 6, 17, 43. Changes in sexual behaviour, immune activation by STIs, direct effects of HIV‐1 infection or bleeding could be involved in the observed HCV susceptibility upon sexual contact 12, 44, 45, 46. Here, we identified an important role for mucosal LCs in allowing HCV entry into mucosal tissues, where the virus remains infectious for transmission by LCs to target cells. The retention of HCV in mucosal tissues by LCs might allow entry of virus into the blood or into lymphoid tissues where LCs might transfer the virus to other cells leading to dissemination to the liver.

Anal intercourse is the primary route for HIV‐1 infection among MSM 47, underscoring the importance of the anal mucosa as entry site for sexually transmitted viruses. Interestingly, LCs are present in sigmoid colon and rectal mucosal tissue observed by immunohistochemistry 26. Furthermore, it has been shown that LCs are present in HIV‐1 negative MSM anal tissue 48, 49, 50. We have identified and quantified LCs in anal mucosa of HIV‐1 positive MSM biopsies. The presence of LCs within mucosal biopsies suggests that LCs are a potential target for HCV during sexual intercourse. Primary LCs were detected in anal mucosal biopsies at similar levels as observed in epidermis, suggesting that mucosal LCs similarly as in skin surveil for invaders. Moreover, mucosal LCs were similar to skin LCs as both subsets expressed high levels of the LC marker CD1a and langerin, whereas they lacked expression of DC‐SIGN. Due to the very low numbers of LCs isolated from anal biopsies, we used primary skin LCs to investigate their role in HCV susceptibility after sexual contact. Throughout our study we used HCV concentrations that were similar to those found in semen of HIV‐1 infected individuals 21, 51, 52. Notably, neither immature nor mature LCs were infected by replication competent HCV. Moreover, activation of mature LCs by TLR ligands did not induce replication of HCV in LCs. Similarly, LCs were not infected by HCV in the *ex vivo* tissue model, and HIV‐1 exposure did not lead to productive HCV infection of LCs *ex vivo*. These data strongly suggest that LCs do not become infected by HCV and therefore infection of LCs is not involved in mucosal HCV dissemination.

Although immature LCs were not able to transmit HCV to hepatocytes, HIV‐1 exposure to LCs or maturation of LCs induced HCV transmission to hepatocytes. Hepatitis C virus transmission by HIV‐1‐exposed or mature LCs was independent of HCV infection as single‐cycle pseudotyped HCV were transmitted by LCs after HIV‐1 exposure. Thus, our data suggest that HIV‐1 infection or activation alters the ability of LCs and allows retention of infectious HCV for transmission to target cells. Mature LCs were very efficient in uptake of HCV and further studies are required to identify the molecular mechanism. HIV‐1 replication is important in HCV retention by LCs, as treating tissues *ex vivo* with different antiretroviral inhibitors abrogated the induced transmission of HCV to hepatocytes. As most MSM living with HIV‐1 are under treatment with cART, these data suggest that either low level of HIV‐1 replication in LCs or activation of LCs by HIV‐1 or co‐infections might result in enhanced HCV susceptibility. Moreover, our data strongly suggest that the maturation state of LCs is involved in HCV transmission as not only HIV‐1 infection but also maturation of LCs enhanced HCV transmission.

It remains unclear whether there is ongoing replication of HIV‐1 within immune cells in cART treated individuals. Some studies have observed low levels of HIV‐1 replication in lymphoid tissue biopsies from infected humans under cART, which was associated with lower antiretroviral drug concentrations in these tissues 53, 54. However, other studies were not able to detect HIV‐1 replication in patients under cART 55, 56, 57. Our data suggest that not only HIV‐1 infection but also tissue activation enhances sexual transmission of HCV.

Immature LCs protect against HIV‐1 infection by inducing langerin‐mediated autophagic degradation of captured HIV‐1 58, whereas maturation of LCs allows infection and subsequent transmission of HIV‐1 to T cells 23, 41, 59. Similarly, our data suggest that immature LCs do not transmit HCV possibly via langerin but, strikingly, maturation of LCs changes this protective behaviour and allows transmission of HCV independent of replication. Thus, maturation of LCs might be important in allowing HCV entry into mucosal tissues.

Several receptors are able to facilitate this so‐called trans‐transmission of HCV to target cells, such as DC‐SIGN and L‐SIGN 24. Interestingly neither immature nor mature LCs express DC‐SIGN or L‐SIGN, suggesting that another receptor might be involved. Heparan sulfates such as Syndecans are important in HCV infection of hepatocytes 60. As these receptors are involved in trans‐transmission of HIV‐1 61, it is possible that this family of receptors also facilitate trans‐transmission of HCV by mature LCs. The ability to transmit HCV might also be associated with lower expression of langerin on mature LCs as langerin has anti‐viral properties 40. Further studies are required to identify the molecular mechanisms that are involved in HCV transmission by mature LCs.

Despite positive treatment outcome after the introduction of successful DAA, MSM are at high risk for HCV reinfection 15, 16. Several factors could account for the increased HCV susceptibility observed in MSM such as more porous rectal mucosa or cellular changes within rectal mucosa 12, 62. The ability by LCs to transmit HCV is not only triggered by HIV‐1 as we also showed that mature LCs transmitted HCV. These data strongly suggest that other STIs as well as mucosal immune activation might enhance HCV susceptibility. Indeed, sexually‐transmitted diseases are very prevalent within the MSM population such as HSV‐2, Chlamydia, HPV infection, gonorrhoea and syphilis 44, 45, 46. Our data strongly suggest that HIV‐1 exposed or mature mucosal LCs efficiently capture and retain infectious HCV. The retention of HCV in mucosal tissues might increase the chance of HCV to enter the blood, or HCV might be transmitted by LCs to lymphocytes in mucosal tissues or after migration into the lymphoid tissues thereby allowing dissemination of HCV. Interestingly, HCV replication has been detected in peripheral blood mononuclear cells, suggesting that these cells might be involved in dissemination 63. Here we have uncovered an important role for primary LCs present in anal mucosa in HCV susceptibility. Further investigation into the efficacy of cART in mucosal tissues might lead to better understanding of the role of HIV‐1 in LC activation.

## 5 Conclusions

Our data strongly indicate that HIV‐1 or immune activation lead to capture and retention of HCV by mucosal LCs, which might facilitate transmission to other cells or entry into the blood. Novel therapies targeting HCV interaction with LCs and abrogation of LC activation by HIV‐1 or other STIs might prevent HCV transmission. This study will help further characterize the biological mechanisms underlying mucosal HCV transmission in MSM living with HIV‐1.

## Competing interest

All authors declare that they have no competing interests.

## Authors’ contributions

B.M.N. performed and designed the research study, analysed the data and wrote the paper. R.S‐F. performed and designed parts the research study. G.S.S. performed parts of the research study and analysed the data. R.R.C.E.S. performed parts of the research study and analysed the data. S.M.K. performed parts of the research. R.M. designed the research study. J.S. designed parts of the research study. P.R. designed parts of the research study. M.L.S.H. contributed essential material. M.V. contributed essential material and designed parts of the research study. C.M.S.R. designed parts of the research study. T.B.H.G. was involved in all aspects of the study.

## Supporting information


**Figure S1.** Gating strategy of phenotyping LCs from anal biopsies.

**Figure S2.** DC‐SIGN is not expressed by primary mucosal and skin LCs.

**Figure S3.** HIV‐1 infection of mature LCs.

**Figure S4.** Mature Langerhans cells and MUTZ‐LCs transmit HCV to hepatocytes.

**Figure S5.** Mature Langerhans cells bind and internalize HCV.
